# Differentiating Resistance Levels and Biochemical Responses of Soybean Cultivars Infected by Diverse *Diaporthe* Species Using Two Inoculation Methods

**DOI:** 10.3390/plants15091284

**Published:** 2026-04-22

**Authors:** Behnoush Hosseini, Kristina Petrović, Jovana Šućur Elez, Marina Crnković, Febina Mathew, Nitha Rafi, Tobias Immanuel Link

**Affiliations:** 1 Department of Phytopathology, Institute of Phytomedicine, Faculty of Agricultural Sciences, University of Hohenheim, Otto-Sander-Str. 5, 70599 Stuttgart, Germany; behnoush.hosseini@uni-hohenheim.de; 2 Breeding Department, Maize Research Institute, 11185 Belgrade, Serbia; 3 Department for Plant Protection, Agriculture Institute of Slovenia, Hacquetova ulica 17, 1000 Ljubljana, Slovenia; 4 Department of Field and Vegetable Crops, Faculty of Agriculture, University of Novi Sad, 21000 Novi Sad, Serbia; marina.crnkovic@polj.uns.ac.rs; 5 Department of Plant Pathology, Microbiology and Biotechnology, North Dakota State University, Fargo, ND 58102, USA; nitha.rafi@ndsu.edu

**Keywords:** soybean, *Diaporthe*, resistance, Stem Cut inoculation, Stem Wound inoculation, relative treatment effect (RTE), biochemical responses

## Abstract

*Diaporthe* spp. are among the most serious pathogens of soybean. Many different *Diaporthe* species can infect soybean plants. The species differ in their aggressiveness or virulence and in the severity of the damage they cause. Resistance breeding in soybean has been performed for only two *Diaporthe* species, so far. It would be very advantageous to identify soybean cultivars with resistance against other *Diaporthe* species as well, both as sources of resistance for breeding and to inform farmers which cultivars should be planted when a given *Diaporthe* species shows high incidence. We performed greenhouse experiments to differentiate levels of resistance using the Stem Cut and Stem Wound methods for inoculation of the plants with *Diaporthe*. Symptom severity was rated visually, and at 5 dpi the level of lipid peroxidation (LP), activity of superoxide dismutase (SOD), total phenolics and total flavonoids were measured. Among the four *Diaporthe* species tested, *D. caulivora* was most aggressive, followed by *D. longicolla*. Of the cultivars evaluated, Magnolia exhibited the highest level of resistance with no significant differences observed among the other cultivars. Although biochemical responses could be observed, it was impossible to determine the specific response responsible for elevated resistance in Magnolia.

## 1. Introduction

Soybean (*Glycine max* (L.) Merr.) is a crop species of enormous importance. It is a good source of both fat and protein and is used for human consumption either fresh or after processing as salad oil, margarine, mayonnaise, tofu, or, recently, also as milk, yogurt, and cheese. The meal fraction from oil production is used as high-protein animal feed for pigs, poultry, and cattle. Together, these make soybean almost indispensable for human nutrition directly or indirectly and, as a legume, soybean can also fix elemental nitrogen, making it very useful in crop rotations, improving soil fertility. Soybean cultivation is concentrated in the Americas and East Asia. The total soybean area in Europe is currently estimated at approximately 5.5 million hectares [1], and within the European Union (EU) it has recently exceeded 1 million hectares, with continued growth expected, in part because of increasing demand for locally produced non-GMA legumes and government support aimed at making Europe independent from protein imports.

Species of genus *Diaporthe*, also known as the *Diaporthe/Phomopsis* Complex [2], can cause several different diseases on soybeans. These are pod and stem blight, seed decay, stem canker, and seedling blight [3]. Together, these diseases are responsible for substantial yield losses worldwide, surpassing the impact of any other pathogen affecting soybeans. The pathogens can be controlled by cultural practices like residue management, extended crop rotation, testing seed against *Diaporthe* spp., and by fungicide application [3]. However, the most effective control strategy is planting resistant soybean cultivars. Therefore, the identification of cultivars resistant to the different species or different strains of *Diaporthe* is highly relevant. These cultivars can either be prioritized in cultivation where *Diaporthe* is a problem or used as a source for resistance in breeding programs.

In Europe, *Diaporthe longicolla* (Hobbs) J.M. Santos, Vrandečić & A.J.L. Phillips and *D. caulivora* (Athow & Caldwell) J.M. Santos, Vrandečić & A.J.L. Phillips are considered the most important pathogens of soybeans within the genus *Diaporthe*. *D. longicolla* is primarily associated with seed decay but can also cause pod and stem blight, often together with *D. sojae* [4]. *D. caulivora* is the causal agent of northern stem canker and is also capable of infecting seeds [5,6,7,8]. *D. aspalathi* E. Jansen, Castl. & Crous, causes southern stem canker [9] and, together with *D. caulivora,* is the only species against which systematic resistance breeding has already been performed [10]. *D. aspalathi* seems to be restricted regionally and has not yet been detected in Europe. Other *Diaporthe* species associated with soybean and capable of infecting and causing disease include: *D. aseana* Dissan., Tangthir. & K.D. Hyde (syn *D. tectonigena* Doilom, Dissan. & K.D. Hyde), *D. bacilloides* K. Petrović, Skaltsas & F.M. Mathew, *D. eres* Nitschke, *D. flavescens* K. Petrović, Skaltsas & F.M. Mathew, *D. foeniculina* (Sacc.) Udayanga & Castl., *D. goulteri* R.G. Shivas, S.M. Thomps. & Y.P. Tan, *D. gulyae* R.G. Shivas, S.M. Thompson & A.J. Young, *D. insulistroma* K. Petrović, Skaltsas & F.M. Mathew, *D. kongii* R.G. Shivas, S.M. Thompson & A.J. Young, *D. masirevicii* R.G. Shivas, L. Morin, S.M. Thomps. & Y.P. Tan, *D. novem* J.M. Santos, Vrandečić & A.J.L. Phillips (syn. *D. pseudolongicolla* K. Petrović, L. Riccioni & M. Vidić) (The species is treated here as *Diaporthe novem*, following current usage in Index Fungorum [11] and Species Fungorum [12]. However, an alternative interpretation recognizing the name *D. pseudolongicolla* has been proposed by Petrović et al. [13].), *D. rudis* (Fr.) Nitschke, *D. ueckeri* Udayanga & Castl. (syn. *D. ueckerae* Udayanga & Castl., *D. miriciae* R.G. Shivas, S.M. Thomps. & Y.P. Tan), and *D. unshiuensis* F. Huang, K.D. Hyde & Hong Y. Li [3,14,15,16,17]. *D. goulteri* was only very recently isolated from soybeans for the first time [18]. So far, not all species of *Diaporthe* are present in all soybean-producing regions, but they are spreading further.

The taxonomy of the genus *Diaporthe* is complex. In recent multilocus phylogenetic studies of the genus, many species names were found to be synonymous, and species boundaries were redefined [19,20]. Many species can infect more than one host and can either behave as pathogens or endophytes, depending on the host and weather conditions. The interactions of different *Diaporthe* species with soybean also widely vary, as can be deduced from the different diseases that are caused. For example, *D. longicolla* can cause pod and stem blight, black zone lines, and seed decay. This is accompanied by the observation that in different conditions, plants may either be killed by seedling blight, stem blight, or stem canker, or appear healthy throughout the growing season and only after the onset of senescence, pycnidia and perithecia appear on the plant surface and seeds may contain *Diaporthe* mycelium [8]. These late symptoms might be due to late infection, but under some conditions, the species behave like endophytes during plant growth and only switch their lifestyle at the onset of plant senescence. Neither possibility can be excluded at present. These things are still not entirely clear, even for the most important pathogens *D. caulivora, D. aspalathi*, and *D. longicolla*, for which genome sequencing has been performed already [9,21,22,23,24,25], and even less is known about the other species associated with soybeans. It is not entirely clear how they penetrate the plant, how they spread through the plant and how, when, and why sporulation is induced.

Given the taxonomic complexity and partially overlapping symptomology of these pathogens, diagnostic procedures were, until recently, limited to identifying the genus *Diaporthe*. This has changed recently. Now, species-specific molecular diagnosis based on real-time PCR is available for several species [10,18,26,27]. Likewise, studies are performed to differentiate the species or isolates of the species based on their virulence against different soybean cultivars or accessions [8,14,28,29,30]. For this, methods have been developed to inoculate soybean plants with *Diaporthe* and time schedules and grading scales for assessing disease severity have been optimized. With a view to obtaining results fast and with a minimal use of resources, assays have been developed where germinating seeds are immersed in a spore suspension [16]. Alternatively, in the greenhouse, seedlings are inoculated on the stem. Here, either the stem can be pierced with a toothpick overgrown with fungal mycelium, or an agar block with *Diaporthe* mycelium is applied to the stem that can also be wounded in different ways [28]. For the latter methods, humidity at the inoculation site and in the greenhouse is critical, since it has a strong influence on whether there is successful infection and can determine the nature of the symptoms that develop after infection.

Building upon these recent advances, the present study evaluates the susceptibility of different early maturing European soybean cultivars against different *Diaporthe* species. We inoculated the eight different soybean cultivars Magnolia, Salsa, Nessie, Tofina, Kofu, Orakel, Selena, and Lela with the four species *D. longicolla*, *D. caulivora*, *D. novem* (syn. *D. pseudolongicolla*), and *D. goulteri*. The susceptibility or resistance and biochemical responses of the cultivars and the virulence of the pathogens were assessed both visually and by measuring biochemical parameters of the infected plants.

## 2. Results

When the experiment was performed first, the eight soybean cultivars Magnolia (MG 00), Salsa (MG 00), Nessie (MG 0), Tofina (MG 00-0), Kofu (MG 000-00), Orakel (MG 0), Selena (MG 0), and Lela (MG 0) were inoculated with one isolate each of *D. longicolla*, *D. caulivora*, *D. novem* (syn. *D. pseudolongicolla*), and *D. goulteri.* The two inoculation methods Stem Cut and Stem Wound were used. In this initial experiment both inoculation methods produced reliable infections under conditions of high humidity. Clear differences in disease severity of the different species × cultivar combinations could be observed. To confirm the results, a repeat experiment was performed. Since the differences in the biochemical parameters appeared to be more pronounced for the Stem Wound method, this method was selected for the repeat experiment and the selection of cultivars was reduced to the five cultivars Kofu, Salsa, Tofina, Magnolia (German cv.), and Selena (Serbian cv.) that had shown the biggest differences in the first experiment. The Stem Cut method was not repeated because, while it yielded similar results, these data could not be integrated into the evaluation of the Stem Wound data.

### 2.1. Visual Evaluation of Disease Severity

Table 1 shows disease ranking and relative treatment effect for eight different cultivars and four different *Diaporthe* species in the experiment using the Stem Cut method.

The RTE was highest for Salsa infected with *D. caulivora,* significantly higher than all other combinations except for Selena and Nessie, also infected with *D. caulivora*. From all combinations a tendency could be observed for severe infections by *D. caulivora* and *D. longicolla*, while *D. goulteri* and *D. novem* seem to be less aggressive. Apparently, the species generally considered the major pathogens from the genus *Diaporthe* are also the most aggressive. However, there are exceptions, for example, in cv. Nessie, *D. goulteri* caused an RTE similar to that of *D. longicolla* and even *D. caulivora*. Since the lowest RTE was shared by several combinations, a most resistant cultivar could not be determined. However, cv. Lela had the lowest RTE for three of the pathogen species and only showed medium RTE when infected with *D. caulivora*. Generally, cv. Magnolia also exhibited lower RTEs. Cultivar Kofu displays an interesting pattern, since it showed moderate but similar RTEs. Seemingly, there is a low level of resistance present against all species in this cultivar. That the rankings differ between *Diaporthe* species and cultivars indicates that there are specific interactions.

Table 2 shows disease ranking and relative treatment effect for five different cultivars and four different *Diaporthe* species in two experiments using the Stem Wound method.

The highest disease severity based on RTE was found for the combinations Salsa × *D. caulivora* (0.79), Tofina × *D. caulivora* (0.77), and Selena × *D. longicolla* (0.72). The infections of Selena × *D. novem* (0.29), Magnolia × *D. goulteri* (0.26), and Kofu × *D. goulteri* (0.21) were least severe. The homogeneity of variances between the two experiments was satisfied using the Fligner–Killeen test (*p* = 0.30), so there was no objection against evaluating the repeats together. In addition, the trends seen with the Stem Cut method were the same as those seen with the Stem Wound method, indicating that the results are stable and reliable.

**Figure 1:**
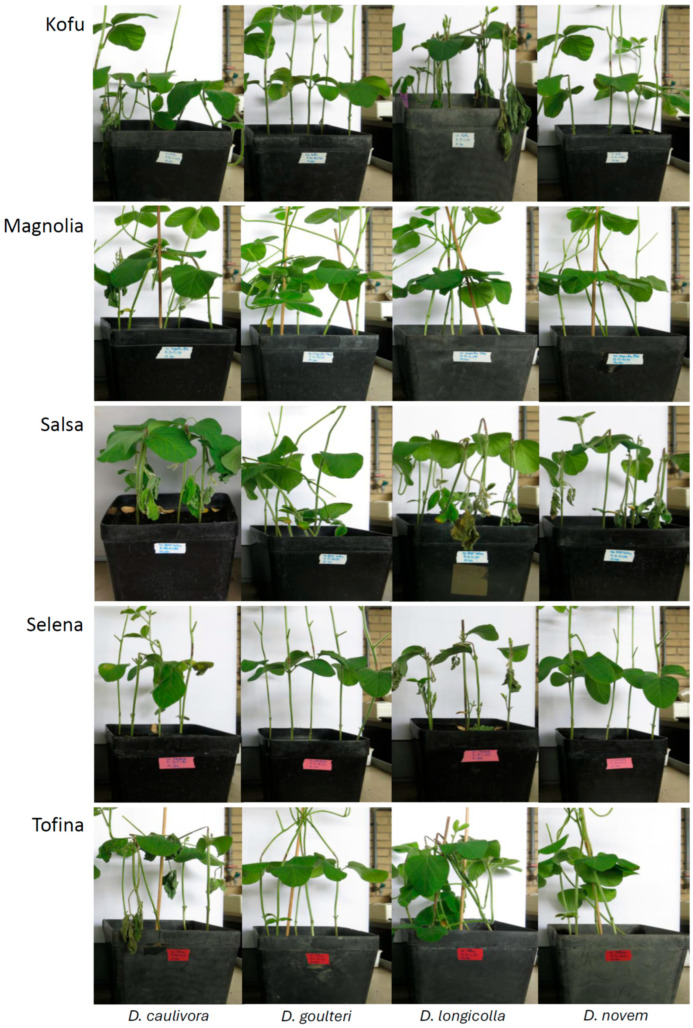
Representative pictures of infected plants five days after Stem Wound inoculation. The soybean cultivar is noted on the left and the *Diaporthe* species is noted below the columns.

Figure 1 shows representative pictures with disease development for all cultivars inoculated with all species.

The data were also analyzed for differences in aggressiveness of the different *Diaporthe* species across all cultivars (Table 3).

Overall, *D. caulivora* was most aggressive, followed by *D. longicolla*. *D. novem* was much less aggressive, but still significantly more aggressive than *D. goulteri*. This nicely aligns with the findings for the specific pathogen on cultivar results, where *D. caulivora* was most aggressive on three cultivars (Magnolia, Salsa and Tofina), while *D. caulivora* and *D. longicolla* were equally most aggressive on two cultivars (Tofu and Selena). Only for one cultivar (Magnolia), *D. novem* and *D. longicolla* showed similar aggressiveness. *D. goulteri* was least aggressive on all five cultivars, whereas *D. novem* was least aggressive on all cultivars except Kofu.

To identify the most resistant cultivar, disease severity was analyzed across all species of *Diaporthe* (Table 4).

In this experiment, cv. Magnolia exhibited the lowest overall disease severity, indicating that this cultivar possesses enhanced resistance against or tolerance towards *Diaporthe* species. Differences between the other four cultivars were small and not statistically significant. When resistance against the individual species is considered, Magnolia showed intermediate values for *D. caulivora* and had the lowest disease severity for *D. longicolla, D. goulteri,* and *D. novem*.

Because of the different rating scales, the symptoms resulting from the two inoculation methods could not be evaluated together, but, nevertheless, they can be compared. Cultivars Lela, Nessie, and Orakel were only tested in the Stem Cut assay, so these were excluded from the comparison. In both assays, the combination Salsa × *D. caulivora* showed the most severe symptoms. Kofu × *D. goulteri*, which showed the mildest symptoms in the Stem Wound assay, displayed higher severity in the Stem Cut assay but was still among the lower-ranked combinations. Although differences between the two assays were observed, the overall patterns were comparable. These differences probably reflect normal biological variation rather than methodological inconsistency. Taken together, these results are clear and robust.

### 2.2. Biochemical Responses of Infected Soybean Plants

**Figure 2:**
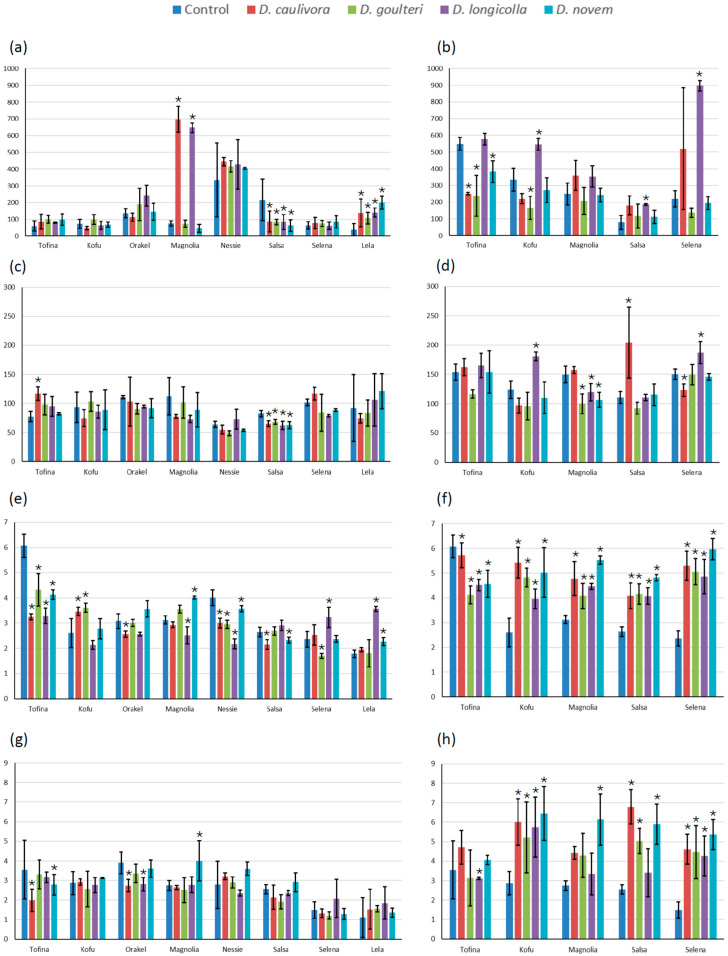
Biochemical parameters illustrating plant responses to *Diaporthe* infection after inoculation using the Stem Wound method. (**a**,**b**) SOD activity (U/g FW), (**c**,**d**) MDA content (nmol MDA/g FW), (**e**,**f**) phenolics content (mg GAE/g DW), (**g**,**h**) flavonoids content (mg QE/g DW); (**a**,**c**,**e**,**g**) first repeat of the experiment; (**b**,**d**,**f**,**h**) second repeat of the experiment. Columns represent the means of three measurements using independent samples, error bars show standard deviations, and asterisks indicate values significantly different from the control. Cultivar names are given on the x-axis and the colors of the columns indicate the *Diaporthe* species.

The visual assessment of symptoms was complemented by measuring biochemical responses in infected plants. The two inoculation methods were evaluated separately. Because the experiment was only repeated for the Stem Wound method, data for the Stem Cut assay are not presented. The two experimental repeats were evaluated independently and are presented separately in Figure 2. Fewer cultivars were included in the second repetition than in the first. The results of the first repetition are shown on the left side of the figure, those of the second repetition on the right.

There was substantial variation between the two repetitions of the experiment. The relative differences between the values were inconsistent between experiments. An example is the values for SOD activity for cv. Selena. In the first repeat, SOD activity was relatively low in all plants, but in the second repeat, it was higher for all plants and considerably higher than for all others in plants infected with *D. longicolla*. Similar inconsistencies were observed for all the parameters studied.

Searching for correlations between disease severity and the biochemical parameters, it seems a good option to compare, in general, Magnolia, the most resistant cultivar, against the other cultivars, and especially Salsa, which appeared to be the most susceptible. Here, it appears that over both experimental repeats, Magnolia has slightly elevated levels of total phenolics and flavonoids. A significant increase in these compounds could not be observed in both repeats, however. This might indicate that phenolics and flavonoids inhibit *Diaporthe* but are not necessarily induced as a response to the infection.

Differentiating between the pathogens, where *D. caulivora* and *D. longicolla* proved most aggressive, the induction of SOD in Magnolia by these two pathogens in the first repeat is most striking. Though much less pronounced, this induction is also visible in the second repeat and can also be observed in the second repeat for Salsa, Selena and (× *D. longicolla*) in Kofu. Since SOD is activated as a response against oxidative stress, it might be assumed that Magnolia shows reduced disease symptoms because it is best able to deal with oxidative stress. Overall, high levels of SOD coincide with high RTSs, however. So, high SOD primarily is an indicator of plant stress.

MDA accumulation is an indicator of damage to cell membranes. At least partially, accumulation of MDA also coincides with disease severity. This shows that severe infections with *Diaporthe* on soybean also lead to increased cell damage.

## 3. Discussion

Our study could show clear and significant differences in virulence or aggressiveness between the different tested *Diaporthe* species based on visually observed symptom severity. These findings are consistent with earlier reports documenting substantial variation in aggressiveness among *Diaporthe* species infecting soybean [5,6,7,8,10,24,25,31]. In both inoculation assays, *D. caulivora* consistently ranked as the most aggressive, followed by *D. longicolla*, whereas *D. novem* (syn. *D. pseudolongicolla*) and *D. goulteri* generally induced only mild to moderate symptoms. This confirms that the species previously associated with major stem and seed decay symptoms (*D. caulivora* and *D. longicolla*) indeed pose the highest pathogenic risk.

Overall, cv. Magnolia displayed lower symptom severity than the other cultivars tested. This was most pronounced for *D. longicolla*. This seems to indicate that there is general resistance against all four species, but stronger resistance, that may be species-specific, against *D. longicolla*. In practical terms, this means that cv. Magnolia can be recommended to farmers when there are problems with *Diaporthe*, especially when there is a high incidence of *D. longicolla*. Possibly, the specific resistance against *D. longicolla* can be used for breeding purposes. In the Stem Cut assay, also cv. Lela performed well. Here, also, the low RTE for *D. longicolla* is conspicuous. Since these results are only based on a single experiment, they are not as solid as those for cv. Magnolia; nevertheless, the same conclusions as for cv. Magnolia might be drawn for cv. Lela.

Our approach, however, has several inherent limitations. First, little is known about how the fungi infect and spread in the plant. Therefore, it is difficult to determine how well artificial inoculation methods represent natural disease progression. To be able to test several combinations of pathogen and cultivar, it is necessary to use a method that requires a minimum amount of work. This is true for the methods using inoculation of soybean plants at the stem. Based on earlier studies comparing these methods [28,32], the Stem Cut and Stem Wound methods were chosen as the most suitable.

A second limitation is the use of only one isolate per species. It is possible that different isolates would have shown different degrees of aggressiveness [30]; therefore, it is not entirely clear whether the differences we observed are general differences between the species or just between the specific isolates. Indeed, scoring plants three months and later after planting and inoculation, Hosseini et al. [7] found differences between *D. caulivora*, *D. longicolla*, and *D. novem* isolates. Nevertheless, in this instance, the scale of this experiment required prioritizing inter-species comparisons. Given greenhouse space and the need to inoculate all plants within a short timeframe to maintain uniform humidity, including multiple isolates was not feasible. For *D. goulteri,* only one isolate was available. The assumption that inter-species differences exceed intra-species variation appears supported by our results; still, future studies using multiple isolates per species would refine species-level aggressiveness profiles. Other studies have compared different isolates of the same *Diaporthe* species [30]. These studies were especially interested in finding differences in the virulence of isolates from different regions. To determine how much of the differences in virulence between different *Diaporthe* sp. isolates are due to different traits of the species and how much are due to interspecies variability, studies would be necessary, encompassing all strains of all tested species while comparing at least two species. This, of course, is impossible; any study can only include a small selection of the available isolates, and these may or may not represent a large part of the variability of a species. As more datasets using comparable procedures become available, meta-analyses may offer deeper insights into pathogenic variability in the *Diaporthe* complex.

The need for testing multiple isolates is also illustrated by a discrepancy of our results for the virulence of *D. novem* (syn. *D. pseudolongicolla*) with findings from Petrović et al. [13]. In contrast to the relatively low RTE indicating little aggressiveness, the *D. pseudolongicolla* isolate of [13] caused extensive pathogenicity on soybean stems using the Stem Wound method. The four isolates tested by Hosseini et al. [7] differed in their disease score. The more recent isolate DPC_HOH41 used in this study is also different. What also should be mentioned here is that, in contrast to *D. longicolla*, where ITS, *TEF1* and *TUB* sequences used for molecular phylogenetic analysis are 100% identical for all isolates, isolates of *D. novem* differ in these marker sequences [7]. Indeed, DPC_HOH41 is also slightly different in those sequences both to the isolates by Hosseini et al. [7] and Petrović et al. [13]. So, it can be speculated that *D. novem* is a species with especially high intraspecific variation.

High MDA levels as a marker for lipid peroxidation and cell membrane damage indicate that the plants are suffering from stress [31]. In their study, Petrović et al. [31] chose an inoculation method they termed “mycelium contact” because, based on MDA levels, this method caused the lowest level of stress. The Stem Wound method used here is most similar to what is labeled as the “plug method” by Petrović et al. [31]. Presumably because of the wounding of the stem that is performed in this procedure, the plants suffer elevated levels of stress from the inoculation procedure. This aligns with our results: in all cultivars except Salsa, MDA levels in control plants are comparable or higher than those in the plants infected with *D. goulteri* or *D. novem*.

Only in cultivars Selena, Kofu, and Salsa, MDA levels in those plants infected with *D. longicolla* rose significantly above the control values. It seems that, though *Diaporthe* infections do cause oxidative stress, cultivars like Salsa that seem to be able to efficiently deal with this kind of stress are not consequently also resistant against the pathogens.

In their study, Petrović et al. [31] studied the activities of superoxide dismutase (SOD), catalase, and peroxidase and the glutathione content, in addition to MDA. The results for these other parameters could not be correlated with tolerance, as well as the MDA level. Therefore, in this study, we only studied SOD again and came to the same conclusion, that SOD is primarily a marker of oxidative state rather than a predictor for host resistance.

Instead of enzyme activities, we studied total phenolics and total flavonoids. Among the phenolics, and especially the flavonoids, are many substances with fungicidal or fungistatic properties. These are either permanently present in plants, contributing to pre-formed resistance as phytoanticipins, or are produced upon induction of plant defenses as phytoalexins. Therefore, elevated levels of phenolics and flavonoids can indicate resistance against fungal pathogens. Since we found Magnolia to be most resistant and Salsa to be most susceptible, it might be expected that Magnolia has comparably high levels of phenolics and flavonoids, and Salsa has low levels. This could be observed, but to a very small extent and not consistently. While in the first repeat, the levels were slightly higher for Magnolia than for Salsa, they were very similar for the two cultivars in the second repeat of the experiment. Moreover, Salsa × *D. caulivora,* with the highest RTE, had unexpectedly high flavonoids accumulation. Thus, it must be concluded that either phenolics and flavonoids do not play a major role in defense against *Diaporthe*, or that certain *Diaporthe* species can suppress or bypass those defense phenolics- and flavonoids-mediated defense pathways, preventing these metabolites from acting as reliable resistance indicators.

Taken together, the biochemical markers measured here did not provide reliable predictors of resistance or susceptibility. Visual disease severity remained the most consistent and biologically meaningful indicator of *Diaporthe* aggressiveness and soybean cultivar response.

These considerations highlight the complexity of *Diaporthe*–soybean interactions and emphasize the need for multi-isolate, multi-cultivar studies using standardized inoculation procedures to better resolve species- and genotype-specific pathogenic behaviors.

## 4. Materials and Methods

### 4.1. Diaporthe Isolates, Soybean Cultivars, and Inoculation Methods

Isolates of the four *Diaporthe* species *D. longicolla*, *D. caulivora*, *D. novem* (syn. *D. pseudolongicolla*), and *D. goulteri* were selected from the Phytopathology lab collection, University of Hohenheim (Table 5). The fungi were sub-cultured on fresh potato dextrose agar (PDA) plates containing autoclaved toothpicks and kept at room temperature (23 °C) for 10 days. To have fresh isolates, two soybean plants were inoculated with each *Diaporthe* isolate using the toothpick method [28,33]. One month after the appearance of symptoms on stems (stem canker or pod and stem blight symptoms), the fungi were re-isolated from the plants. Small pieces of the stems were disinfected using 1% sodium hypochlorite for 30 s, rinsed three times with sterile distilled water, dried on filter paper, and then cultured on PDA and kept at 23 °C for 3 days. After that, agar blocks with fresh mycelium were taken from the margin of the plates and transferred to PDA plates. These were incubated for 5 days.

The selected cultivars represent commercially important soybean genotypes in Germany and Serbia, particularly adapted to these regions due to their early maturity. The cultivars, belonging to different early maturity groups (MG, standard European classification), Magnolia (MG 00), Salsa (MG 00), Nessie (MG 0), Tofina (MG 000), Kofu (MG 000-00), Orakel (MG 0), Selena (MG 0), and Lela (MG 0) were kindly provided by the State Seed Breeding Institute of the University of Hohenheim (Stuttgart, Germany) and Maize Research Institute Zemun Polje (Belgrade, Serbia). For each cultivar, 26 pots (5L) containing standard soil (Gebrüder Patzer GmbH & Co. KG, Sinntal, Germany) were prepared and five seeds were put into each pot. Plants were grown in the greenhouse at 16 h light/8 h dark (24 °C/22 °C).

The two inoculation methods, Stem Cut or cut-seedling [32] and Stem Wound [28], were applied to infect the plants with *Diaporthe* isolates. For each cultivar × pathogen combination, 15 plants (three pots) at the V2 growth stage were inoculated.

**Figure 3:**
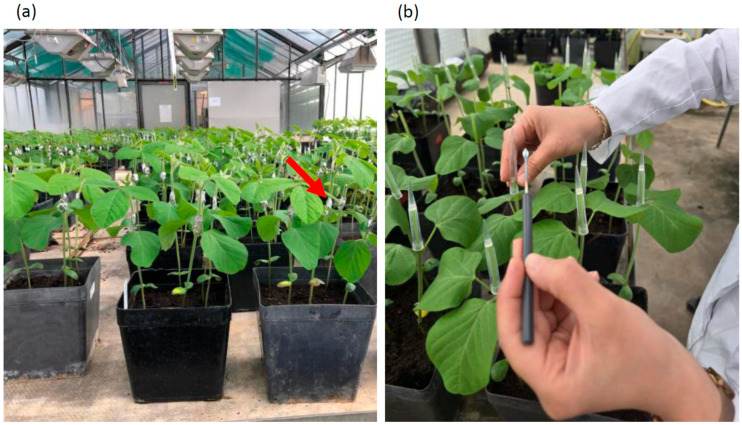
Inoculation of soybean plants with *Diaporthe*. (**a**) Plants freshly inoculated with the Stem Wound method. Inoculation sites (arrow indicates an inoculation site) covered with cotton and aluminum foil. (**b**) Inoculation following the Stem Cut method.

For inoculation with the (modified) Stem Cut method [32], the stems were cut off ~2 cm above the node of the primary leaves and an agar plug with mycelium (~5 mm diameter) was placed on top of the remainder of the stems so that the mycelium was in contact with the stem. To retain the moisture at the inoculation site, a 1000 µL pipette tip was placed on the stem (Figure 3b).

For inoculation using the Stem Wound method [28], a 1 cm long wound was created on the stem midway between the primary leaves and the first trifoliate leaves by scratching the stem with a scalpel. An agar plug with mycelium (~5 mm diameter) was placed on this wound, and to fix it there and keep it moist, a piece of wet cotton and a small strip of aluminum foil were wrapped around the stem at the same position (Figure 3a).

For each inoculation method and each cultivar, five plants (one pot) were inoculated with agar plugs without mycelium as a control. The inoculated plants were kept under transparent plastic foil with high humidity for five days, then they were returned to standard greenhouse conditions. At the same time, the pipette tips were removed from the Stem Cut plants and the aluminum foil and cotton pieces from the Stem Wound plants.

### 4.2. Assessment of Disease Severity

Disease development was first assessed seven days after inoculation and, additionally, 14 dpi, 21 dpi, and 28 dpi (first experiment), and 14 dpi, 21 dpi, 28 dpi and 35 dpi (second experiment). Plants were appraised visually, and symptoms were graded individually for every plant based on the scales presented in Table 6 and Table 7.

The scales differ because breaking of the stems could not be a criterion with the Stem Cut method, where the stems were already cut.

### 4.3. Sampling and Measurement of Biochemical Parameters

Sampling was performed 5 dpi directly after removal of the foil for keeping humidity and the pipette tips or cotton and aluminum strips. From plants, inoculated using the Stem Wound method, the first trifoliate leaf was used. Plants inoculated using the Stem Cut method only had the two primary leaves, one of which was used. The leaves were cut using scissors. For storage and transport, the leaves were either dried for a week at room temperature between sheets of copy paper in a stack of paper towels that were replaced twice during the process or kept frozen in plastic bags (one bag per sample) at −14 °C. While the virulence assay was performed in the facilities of the University of Hohenheim, the leaves were shipped to the University of Novi Sad Faculty of Agriculture for the biochemical measurements.

The dried leaves (0.2 g) were extracted with 70% ethanol (10 mL). After 24 h, the extracts were filtered through filter paper and used for the determination of total phenolics and total flavonoids. Total phenolics were determined according to the Folin–Ciocalteu method [34]. The leaf extracts were mixed with deionized water, 20% sodium carbonate, and Folin–Ciocalteu reagent diluted with distilled water in a proportion of 1:2. The absorbance of the reaction mixture was measured after incubation at room temperature for 30 min, at 720 nm. The data were expressed as mg gallic acid equivalent per gram dry weight (mg GAE/g DW). Total flavonoids were estimated according to the method described by Markham [35]. The leaf extracts were mixed with deionized water (1 mL) and aluminum chloride hexahydrate (2.5 mL). After incubation at room temperature for 15 min, the absorbance of the reaction mixture was measured at 430 nm. The data were expressed as mg quercetin equivalents per g dry weight (mg QE/g DW).

Fresh (frozen) plant leaves (2 g) were crushed and homogenized in phosphate buffer (0.1 M, pH 7.0, 10 mL). After centrifugation, clear supernatants were used for the determination of the lipid peroxidation (LP) intensity and activity of superoxide dismutase (SOD). The intensity of the LP was determined using the thiobarbituric acid (TBA) test at 532 nm [3]. The extract was incubated with 20% trichloroacetic acid (TCA) containing 0.5% thiobarbituric acid for 40 min at 95 °C. The reaction was stopped by cooling on ice for 10 min, after which the product was centrifuged at 10,000 rpm for 15 min. The total amount of TBA-reactive substances was given as nmol of malondialdehyde (MDA) equivalents per gram of fresh weight (nmol MDA/g FW). The assay of SOD activity [36] is based on the ability of the enzyme extracts to inhibit the photochemical reduction in the nitro blue tetrazolium (NBT) chloride. The reaction mixture (50 mM phosphate buffer (pH 7.8), 75 μM NBT, 13 mM L-methionine, 0.1 mM EDTA, 2 μM riboflavin, and enzyme extract) was kept under a fluorescent lamp for 15 min, and the absorbance was read at 560 nm. One unit of SOD activity was defined as the amount of enzyme required to inhibit the reduction in NBT by 50%. The activity of SOD was expressed as U per g fresh weight (U/g FW).

### 4.4. Statistical Analysis

Ordinal disease severity data were analyzed as described in [28] using non-parametric methods for longitudinal data following the procedures of Shah & Madden [37] and Brunner et al. [38]. For each cultivar × pathogen combination, a relative treatment effect (RTE) was computed as “

(1) $${\bar{R}}_{i}=\frac{1}{n_{i}}\sum_{k=1}^{n_{i}}R_{\mathrm{ik}},$$

where $\bar{R}$_i_ = the mean rank for the ith treatment and *R*_ik_ = the rank of X_ik_ among all N observations” [28], representing the probability that a randomly selected plant from that treatment exhibits more severe symptoms than a randomly selected plant from the entire dataset. Specifically, lower RTE values indicate lower disease severity and higher RTE values indicate greater disease severity in comparison to the susceptible variety. Differences among treatments were assessed using ANOVA-type statistic (ATS), and significant interactions were further explored through post hoc pairwise comparisons of RTE values based on the Brunner–Munzel approach with appropriate corrections for multiple testing. All analyses were conducted in R (version 4.2.3.) using the nparLD package [39].

Biochemical parameters (SOD, MDA, total phenolics, total flavonoids) were analyzed using one-way ANOVA followed by Tukey’s HSD post hoc test (*p* ≤ 0.05). Data are presented as means ± SD of three biological replicates.

## 5. Conclusions

Our approach provided clear information about differences in aggressiveness of *Diaporthe* species and resistance to *Diaporthe* of different soybean cultivars. *Diaporthe caulivora* was confirmed as the most aggressive species, followed by *D. longicolla*, while *D. novem* (syn. *D. pseudolongicolla*) and *D. goulteri* showed comparatively low virulence. Since none of the tested cultivars has been subject to breeding efforts for resistance against *Diaporthe* species, it is reasonable to assume that the genetic source for resistance displayed by cv. Magnolia is yet unknown and can be exploited in the future.

The inoculation methods applied here, Stem Cut and Stem Wound, proved to be reliable, reproducible, and suitable for screening large numbers of soybean genotypes. Expanding the evaluation to a broader range of cultivars and isolates will be important for identifying additional resistance sources and for gaining a deeper understanding of *Diaporthe*–soybean interactions.

## Figures and Tables

**Table 1:** Disease rank (mean) and relative treatment effect (RTE) for the experiment using the Stem Cut method. Higher numbers indicate higher disease severity; numbers in brackets indicate the confidence intervals calculated for the RTEs at *p* ≤ 0.05; * indicates the highest disease severity for each cultivar; 15 plants were evaluated for every cultivar × pathogen combination; the experiment was not repeated.

	*D. caulivora*		*D. goulteri*		*D. longicolla*		*D. novem*	
	Rank	RTE	Rank	RTE	Rank	RTE	Rank	RTE
Kofu	334.1	0.70 (0.57, 0.80) *	237.4	0.49 (0.38, 0.61)	188.2	0.39 (0.34, 0.45)	188.2	0.39 (0.34, 0.44)
Lela	247.1	0.51 (0.40, 0.62) *	174.5	0.36 (0.35, 0.38)	174.5	0.36 (0.35, 0.38)	174.5	0.36 (0.35, 0.38)
Magnolia	346.8	0.72 (0.57, 0.83) *	174.5	0.36 (0.35, 0.38)	229.3	0.48 (0.38, 0.57) *	174.5	0.36 (0.35, 0.38)
Nessie	388.6	0.81 (0.65, 0.90) *	296.3	0.62 (0.48, 0.73) *	278.5	0.58 (0.46, 0.69) *	174.5	0.36 (0.35, 0.38)
Orakel	278.5	0.58 (0.46, 0.69) *	174.5	0.36 (0.35, 0.38)	215.6	0.45 (0.36, 0.54)	174.5	0.36 (0.35, 0.38)
Salsa	437.4	0.91 (0.89, 0.93) *	201.9	0.42 (0.35, 0.50)	243	0.51 (0.40, 0.61)	174.5	0.36 (0.35, 0.38)
Selena	402.9	0.84 (0.76, 0.89) *	174.5	0.36 (0.35, 0.38)	255.2	0.53 (0.41, 0.65)	174.5	0.36 (0.35, 0.38)
Tofina	285.5	0.59 (0.43, 0.74) *	174.5	0.36 (0.35, 0.38)	372.9	0.78 (0.68, 0.85) *	174.5	0.36 (0.35, 0.38)

**Table 2:** Disease rank (mean) and relative treatment effect for the experiments using the Stem Wound method. Higher numbers indicate higher disease severity; numbers in brackets indicate the confidence intervals calculated for the RTEs at *p* ≤ 0.05; * indicates the highest disease severity for each cultivar; 2 × 15 plants were evaluated for every cultivar × pathogen combination in the two repetitions of the experiment.

	*D. caulivora*		*D. goulteri*		*D. longicolla*		*D. novem*	
	Rank	RTE	Rank	RTE	Rank	RTE	Rank	RTE
Kofu	327.7	0.60 (0.50, 0.70) *	114.2	0.21 (0.16, 0.27)	372	0.69 (0.59, 0.77) *	284	0.52 (0.42, 0.63) *
Magnolia	331.6	0.61 (0.55, 0.67) *	141.5	0.26 (0.21, 0.32)	188.9	0.35 (0.27, 0.44)	196	0.36 (0.31, 0.42)
Salsa	429.1	0.79 (0.72, 0.85) *	192.2	0.35 (0.28, 0.44)	311	0.57 (0.48, 0.66)	284	0.52 (0.44, 0.61)
Selena	317.9	0.59 (0.51, 0.66) *	196	0.36 (0.30, 0.43)	392.1	0.72 (0.64, 0.80) *	161.9	0.29 (0.24, 0.36)
Tofina	419.3	0.77 (0.72, 0.82) *	196	0.36 (0.29, 0.44)	351.9	0.65 (0.59, 0.71)	202.6	0.37 (0.31, 0.44)

**Table 3:** Disease rank (mean) and relative treatment effect for the experiments using the Stem Wound method. Higher numbers indicate higher disease severity; numbers in brackets indicate the confidence intervals calculated for the RTEs at *p* ≤ 0.05; ^a, b, c, d^ indicate significantly different values; 2 × 75 plants were evaluated for every pathogen in the two repetitions of the experiment.

Pathogen	Mean Rank	RTE
*D. caulivora*	365.1	0.68 (0.65, 0.70) ^a^
*D. goulteri*	168	0.31 (0.28, 0.34) ^d^
*D. longicolla*	323.2	0.60 (0.56, 0.63) ^b^
*D. novem*	225.7	0.42 (0.39, 0.45) ^c^

**Table 4:** Disease rank (mean) and relative treatment effect for the experiments using the Stem Wound method. Higher numbers indicate higher disease severity; numbers in brackets indicate the confidence intervals calculated for the RTEs at *p* ≤ 0.05; ^a, b^ indicate significantly different values; 2 × 60 plants were evaluated for every cultivar in the two repetitions of the experiment.

Cultivar	Mean Rank	RTE
Kofu	274.5	0.51 (0.46, 0.55) ^a^
Magnolia	214.5	0.40 (0.36, 0.43) ^b^
Salsa	304.1	0.56 (0.52, 0.60) ^a^
Selena	267	0.49 (0.45, 0.53) ^a^
Tofina	292.5	0.54 (0.51, 0.57) ^a^

**Table 5:** *Diaporthe* species and isolates used for the inoculation of soybean plants. Isolate DPC_HOH2 is also described in [7], and isolate DHP_HOH36 is described in [18]. DPC_HOH40 and DPC_HOH41 are more recent isolates. All isolates were originally isolated from soybean plants or seeds.

Species	Isolate No.	Origin	GenBank Accessions		
			*ITS*	*TEF1*	*TUB*
*D. longicolla*	DPC_HOH40	Germany	PV669840	PV683073	PV683075
*D. caulivora*	DPC_HOH2	Austria	MK024677	MK099094	MK161476
*D. novem*	DPC_HOH41	Germany	PV669841	PV683074	PV683076
*D. goulteri*	DPC_HOH36	Germany	PQ008930	PQ014381	PQ014385

**Table 6:** Rating scale for the Stem Wound method.

Grade	Description
0	healthy plant (no lesion beyond the wound—1 cm)
1	lesion present but shorter than 2 cm
2	lesion longer than 2 cm, but stem unbroken
3	lesion longer than 2 cm and stem broken
4	stem broken, tissue dead above the inoculation site
5	dead plant

**Table 7:** Rating scale for the Stem Cut method.

Grade	Description
0	healthy plant (lesion shorter than 1 cm)
1	lesion longer than 1 cm but stopping before the primary leaf node
2	lesion extending beyond the primary leaf node
3	lesion extending far (more than 2 cm) beyond the primary leaf node
4	dead plant

## Data Availability

The original contributions presented in this study are included in the article. Further inquiries can be directed to the corresponding authors.

## Abbreviations

The following abbreviations are used in this manuscript:

ATS	ANOVA-type statistics
cv.	cultivar
dpi	days post inoculation
DW	dry weight
FW	fresh weight
GAE	gallic acid equivalent
LP	lipid peroxidation intensity
MDA	malondialdehyde equivalents
MG	maturity group
NBT	nitro blue tetrazolium
QE	quercetin equivalents
PDA	potato dextrose agar
RTE	relative treatment effect
SOD	superoxide dismutase
